# Follow-up of neonatal chronic respiratory disease: an update based on the current evidence

**DOI:** 10.1007/s00431-025-06713-5

**Published:** 2026-01-07

**Authors:** Allan Jenkinson, Theodore Dassios

**Affiliations:** 1 Department of Women and Children’s Health, School of Life Sciences, Faculty of Life Science and Medicine, King’s College London, London, UK; 2 Neonatal Intensive Care Unit, University of Patras, Patras, Greece; 3 Neonatal Intesive Care Centre, Golden Jubilee Wing, King’s College Hospital, Denmark Hill, London, SE5 9RS UK

**Keywords:** Bronchopulmonary dysplasia, Lung function, Pulmonary hypertension, Childhood, Exercise limitation

## Abstract

Chronic respiratory morbidity following premature birth is associated with significant and long-standing complications affecting airway function, the lung parenchyma and cardiac morphology. The aim of this narrative review was to provide an update on the current evidence for the cardiorespiratory follow-up of extremely preterm infants, particularly those with bronchopulmonary dysplasia. There is currently wide variation in the frequency, components and duration in the follow-up of these infants and an absence of robust evidence to support specific recommendations. Follow-up is most commonly offered up to 2 years of age, and studies beyond this age primarily report spirometric indices to quantify lung function impairment and abnormal respiratory trajectories. Recent evidence has highlighted distinct phenotypes and the complex multisystem nature of respiratory disease following preterm birth, which includes patterns of persistent pulmonary vascular disease, abnormal parenchymal function and impaired cardiorespiratory fitness.

*Conclusion*: We suggest that follow-up of these infants should extend in time to early adulthood and focus on capturing and describing not only lung function test abnormalities but also include pulmonary vascular disease, parenchymal disease and cardiorespiratory exercise testing with a view to identify individuals who would benefit most from targeted interventions based on the dominant pathophysiological process at an individual level.

**Table:** 

**What is Known:** • *Preterm birth is associated with significant respiratory morbidity lasting into childhood, adolescence, and adulthood.* • *There is a lack of sufficient high-level evidence to inform how these infants should be followed up in childhood and beyond.* **What is New:** • *Other than lung function abnormalities, preterm-born children and adults have parenchymal lung damage, pulmonary vascular disease and exercise limitation.* • *Follow-up of individuals with significant neonatal respiratory disease should expand beyond early childhood and include more extensive cardiorespiratory assessments.*

**What is Known:**

• *Preterm birth is associated with significant respiratory morbidity lasting into childhood, adolescence, and adulthood.*

• *There is a lack of sufficient high-level evidence to inform how these infants should be followed up in childhood and beyond.*

**What is New:**

• *Other than lung function abnormalities, preterm-born children and adults have parenchymal lung damage, pulmonary vascular disease and exercise limitation.*

• *Follow-up of individuals with significant neonatal respiratory disease should expand beyond early childhood and include more extensive cardiorespiratory assessments.*

## Introduction

Advances in obstetric and neonatal care have resulted in increased incidence of preterm birth [1]. Furthermore, with the decrease of the border of viability to 22 weeks of gestation, the burden of prematurity is expected to continue to increase [2]. Despite recent advances, the incidence of chronic respiratory disease following premature birth, broadly captured by the term bronchopulmonary dysplasia (BPD), continues to rise [3]. The lifelong cardiorespiratory trajectories after preterm birth remain understudied [4, 5]. The first large groups of studied survivors have only recently reached adulthood with emerging evidence of early chronic obstructive pulmonary disease (COPD) and persistent pulmonary hypertension [6, 7]. Despite these lifelong adverse outcomes, clinical follow-up of this population is often limited to 2 years of age, mirroring the 2-year endpoint for research studies, which was proposed in the 1990 s [8]. This narrative review aimed to provide an update on current evidence in the cardiorespiratory follow-up of extremely preterm infants, particularly those with BPD.

## Methods

Manuscripts were identified through a search of PubMed for articles published in English between January 2000 and September 2025. The following search terms were used (‘Bronchopulmonary dysplasia’ OR ‘chronic lung disease’ OR ‘neonatal respiratory disease’ OR ‘extremely preterm’ OR ‘prematurity’) AND (‘lung’ OR ‘pulmonary’ OR ‘exercise’ OR ‘cardiac’ OR ‘cardiopulmonary’ OR ‘cardiorespiratory’) AND (‘long term’ OR ‘follow up’ OR ‘monitor’ OR ‘outcome’). Articles were also identified through searches of references and authors’ libraries. The final list of cited articles was selected on the basis of their relevance to the aims of this review, with a focus on articles published after 2015.

## Guidelines, frameworks and consensus statements

A recent large international survey of neonatologists, paediatricians and pulmonologists highlighted a significant knowledge gap regarding best practice in the follow-up of individuals born preterm [9]. Few reports have evaluated follow-up programmes for children born prematurely, with the available evidence demonstrating that follow-up often ceases at 2 years of age [9, 10]. Despite some heterogeneity in eligibility criteria and duration, the number of European countries with follow-up frameworks increased from 6 to 11 between 2011 and 2022 [10]. The focus of these programmes is typically neurodevelopmental, with only two countries (Sweden and Denmark) reporting respiratory function beyond 2 years and another two (Poland and Italy) specifically mentioning cardiac follow-up.

The European Respiratory Society (ERS) [11] and the American Thoracic Society (ATS) [12] have published clinical guidelines on the follow-up of infants with a history of neonatal respiratory disease. The ERS guideline focused on infants with BPD, defined according to the 2001 workshop [13, 14]. ATS broadened their recommendations to “post-prematurity respiratory disease” which describes all respiratory disease after any preterm birth [15]. The recommendations of both task forces were limited by low-quality evidence. The need for an international consensus on how to structure respiratory follow-up remains largely unmet [16, 17]. The BPD Collaborative consists of an international group of tertiary centres dedicated to improving the care of individuals with BPD [18]. Although the group performed a Delphi study and achieved a consensus on follow-up practices in moderate-severe BPD [19], a subsequent survey of participating centres demonstrated significant inter-centre variability in referral patterns to outpatient clinics, indications for echocardiography at follow-up, availability of lung function testing and criteria for discharge from follow-up [20].

Unlike previously published guidelines and consensus statements, a recent cardiorespiratory follow-up consensus from Canada was the first to recommend *lifelong* follow-up, emphasising the need for comprehensive lung assessment beyond spirometry, evaluation of pulmonary vascular disease and monitoring of exercise outcomes [21].

## Classification and incidence of BPD

A widely accepted consensus definition for BPD was established in 2001 through a National Heart, Lung and Blood Institute (NHLBI) workshop and validated in 2005 [14, 22]. This definition was proposed 25 years ago when survival rates of extremely preterm infants were lower [23, 24]. Newer BPD classifications offer a broader categorisation [23, 25, 26]. Jensen et al. have proposed a new classification for BPD [25] that takes into consideration newer practices such as high-flow nasal cannula therapy [25]. In addition, incorporation of respiratory outcomes beyond the neonatal period in these new classification systems reflects the growing recognition of the long-term effects of prematurity on lung health [13, 25].

Despite advances in neonatal care in the current, post-surfactant era, the incidence of BPD has continued to rise, which likely reflects increased survival at lower gestations [3]. A systematic review documented global variation, with the incidence of moderate BPD ranging from 13 to 31% and severe BPD from 17 to 42% in extremely low birth weight infants [27]. In a recently published single-centre analysis, the incidence of BPD was compared in two birth epochs (2012–2014 and 2020–2022) in the post-surfactant era [28]. Over this period, the incidence of moderate-severe BPD increased from 39 to 64%, likely reflecting more immature and growth-retarded infants in the latter epoch [28].

## Progression to COPD

Although there is some ongoing scientific debate on whether adult BPD constitutes a true subset of COPD, individuals born prematurely are thought to be at increased risk of developing early COPD [29]. The precise underlying pathogenetic mechanisms of this phenomenon are not completely understood but might be related to specific T-cell subset patterns [30], while thickened basement membranes with lymphocytic infiltrates and signs of immature neoangiogenesis have also been reported [31]. Irrespective of the exact pathophysiology, COPD is a life-limiting disorder with irreversible airflow limitation, with airway and alveolar abnormalities [32]. Given that COPD is generally preventable and treatable [33], the implementation of appropriate screening protocols, early detection and timely initiation of preventive and therapeutic interventions are critical to mitigate disease progression and optimise long-term outcomes.

## Monitoring of lung function

In health, lung function follows an established trajectory through the lifespan, increasing during childhood and adolescence and reaching a peak in the early-to-mid-20 s, followed by a gradual physiological decline [34]. Compared with term-born populations, preterm-born individuals are at risk of abnormal trajectories with reduced peak lung function and increased rates of decline, placing them at increased risk for early symptomatic respiratory disease [4].

### Spirometry

Recent meta-analyses have presented comprehensive aggregate data on long-term lung function following preterm birth [35–37]. To date, spirometry remains the gold standard lung function test in this population. In a meta-analysis of studies across all ages with term-born controls including 5501 preterm and 12,648 term individuals, preterms had an FEV_1_/FVC ratio that was 0.56 standard deviations lower than term-born controls [37]. BPD status had a significant impact on airflow obstruction, corresponding to a reduction in the FEV_1_/FVC of 0.87 standard deviations compared to term controls. Meta-regression demonstrated that for every year of older age at assessment, the FEV_1_/FVC ratio of infants with BPD was lower by 0.04 standard deviations. The same study reported that a lower gestational age was associated with a greater lung function deficit [37]. Subgroup analysis demonstrated that individuals born before 28 weeks had the most substantial airflow obstruction with FEV_1_/FVC values of 0.72 standard deviations below term controls. These results are consistent with previously reported data on expiratory airflow limitations in preterm cohorts [38, 39].

Extremely preterm-born individuals, born in the post-surfactant era, have only recently approached adulthood. One limitation, thus, of the above meta-analyses [36, 37, 39] is that they typically report pre-surfactant era adults or cohorts with incomplete surfactant and antenatal corticosteroids [40–43]. Further longitudinal studies of adult cohorts in the post-surfactant era are needed to advance our understanding of lung function trajectories in the current era.

Characterising abnormal spirometry phenotypes may help to predict lung function trajectories and identify therapeutic targets [44, 45]. The proposed phenotypes include prematurity-associated obstructive lung disease, prematurity-associated preserved ratio of impaired spirometry and prematurity-associated dysanapsis [46, 47].

### Oscillometry, plethysmography, gas transfer, inert gas washout

Total body plethysmography and inert gas washout assess static lung volumes indicating restrictive lung disease, gas trapping and ventilation inhomogeneity [48]. The diffusing capacity of the lungs for carbon monoxide (*D*_LCO_) assesses gas transfer indicating alveolar surface area permeability and pulmonary capillary blood volume [49]. In fact, impaired diffusion might better capture the main pathophysiological respiratory phenomenon following premature birth, which is arrested alveolarisation. This process might also be captured by functional indices of decreased alveolar surface area, which are affected in evolving BPD but have only been reported up to 2 years of age [50, 51].

Oscillometry can evaluate indices of respiratory mechanics such as the resistance and compliance of the respiratory system [52]. Oscillometry holds promise in the assessment of the peripheral lung, including the smaller airways that are often labelled “the silent zone” but are increasingly recognised as an important indicator of early lung disease [53].

The first overview of static lung volumes, gas transfer and oscillometry in survivors of preterm birth has recently been published [54]. Their meta-analysis demonstrated that extremely preterm infants and those with BPD had higher residual volume, higher residual volume to total lung capacity and an increased functional residual capacity signalling pulmonary hyperinflation. Reduced gas transfer, as measured by *D*_LCO_ and the transfer coefficient for carbon monoxide, was observed in those born preterm compared to term controls [55].

The forced oscillatory technique (FOT) is a non-invasive, effort-independent, reproducible method for respiratory oscillometry, particularly suitable for repeated measurements in the outpatient setting. While spirometry is generally unsuitable for children under 6 years [56], FOT is feasible in all ages, including neonates, infants and toddlers, and can assess airway obstruction, bronchodilator response and bronchial hyper-responsiveness, as demonstrated in paediatric populations with asthma and cystic fibrosis [57, 58]. Furthermore, the sensitivity of FOT in detecting peripheral airway dysfunction may exceed that of traditional spirometry, making it promising for the diagnosis of lung function phenotypes and temporal monitoring [57, 59].

Extremely preterm cohorts have increased total airway resistance and small airways resistance compared to term controls, indicating altered respiratory mechanics and small airways disease, with greater disparities associated with extreme preterm birth and BPD [60–63]. There is only one longitudinal study of FOT in prematurely born children and young people [64], reporting that among children born at 24–31 weeks of gestation assessed between 6 and 8 [65] and 13 to 17 years of age [64], there were higher resistance values in the BPD compared to the non-BPD group, correlating with worsening FEV_1_/FVC z-scores.

### Lung imaging

The ‘new-BPD’ is characterised by an arrest of alveolar development in the saccular and canalicular stages, which disproportionately affects the peripheral airways [13, 14, 66, 67]. While spirometry and FOT can provide a functional assessment of the peripheral airways, they may not be suitable to evaluate the structure of the distal lung [68]. There is increasing interest in the role of lung imaging as an adjunct to functional assessment in the follow-up of preterm infants [4]. This is not yet reflected in the current ERS/ATS recommendations, which recommend lung imaging only for severe BPD, or for assessing tracheobronchomalacia as a second-line alternative to bronchoscopy [11, 12].

Chest computed tomography (CT) or magnetic resonance imaging (MRI) can detect structural abnormalities and architectural distortion of the distal airways and the lung parenchyma [61, 69, 70]. Evaluation of these changes could help assess phenotypic variations in preterm-born individuals, and combined with functional assessments, they could help identify infants at risk of developing COPD [71]. Chest CT is a sensitive imaging technique for structural lung abnormalities in children [72, 73]. Scoring systems to quantify lung damage have been proposed in BPD, though no method has been validated yet [69]. While it is anticipated that artificial intelligence might improve accuracy, manual scoring currently outperforms automated methods [72]. Radiation exposure remains a significant limitation of CT for longitudinal follow-up [72, 74].

Magnetic resonance imaging (MRI) offers a radiation-free alternative imaging modality and might allow for a more widespread adoption of longitudinal imaging [75]. MRI scoring can quantify interstitial and airway remodelling, emphysematous changes and ventilation inhomogeneity and thus may prove useful as an end-point assessment of targeted treatments or for longitudinal follow-up [76].

Emerging MRI technologies incorporate functional analysis of pulmonary ventilation and perfusion and have been used extensively in the longitudinal assessment in cystic fibrosis. Indirect functional assessment has been demonstrated using T2-weighted lung MRI and dynamic mode decomposition MRI [77–79]. Direct functional assessments using hyperpolarised ^3^He or ^12^Xe are capable of assessing regional ventilation and gas exchange [80]. Although currently confined to research applications due to cost, complexity and lack of validation, they offer significant potential for longitudinal monitoring of ventilation heterogeneity and early detection of microstructural impairment [81]. However promising, CT and MRI are currently highly specialised methods that are resource-intensive, which require specialised equipment, trained personnel and considerable testing time, restricting their general applicability.

## Monitoring of pulmonary vascular disease

Recent evidence has demonstrated that pulmonary vascular disease (PVD) and pulmonary hypertension (PH) are complications of prematurity, with clinical implications extending beyond infancy. Diverse pulmonary vascular phenotypes may present after preterm birth resulting from maladaptive transition, vascular remodelling or injury [82–85]. Infants with BPD are particularly at risk, with abnormal markers of PH that persist throughout infancy [86], childhood [87] and early adulthood [7, 88]. A recent meta-analysis confirmed the association of PH with severe lung disease, with a prevalence of PH in 6%, 12% and 39% of infants with mild, moderate and severe BPD, respectively [89]. In the context of BPD, PH is associated with increased morbidity, including longer duration of oxygen therapy, suboptimal growth, worse neurodevelopment [90] and increased mortality, particularly in the first 6 months post-discharge [91, 92].

The pathogenesis of PVD in preterm infants is multifactorial. In a prospective cohort study of preterm infants, it was demonstrated that ultrasonographic evidence of PVD before 7 days was associated with a subsequent diagnosis of severe BPD and PH [82]. The American Heart Association, the ATS [93] and the European Paediatric Pulmonary Vascular Disease Network [94] have provided guidelines for the diagnosis, monitoring and care of paediatric PH, of which BPD-PH is a recognised subtype. There are no clear recommendations, however, on the follow-up of BPD-PH beyond discharge and no screening recommendations for individuals at risk.

Recent population-based studies from Japan and Sweden have demonstrated the increasing incidence of lifetime PH in individuals born preterm. Data from the Neonatal Research Network of Japan demonstrated that the prevalence of PH among 12,954 extremely preterm infants was 8.1% [95, 96]. This increased annually, with those born at earlier gestational ages being more significantly affected (18.5% for infants born at 22 weeks versus 4.4% for those born at 27 weeks) [95]. In Sweden, a population register study reported that in children and adults with PH, prematurity was associated with an overall increased adjusted risk (odds ratio, 4.48) [96]. Over each 5-year period from 1973, the risk of PH increased, with children born in the 2000 s and later being most affected [96].

Screening echocardiography for high-risk patients in childhood has demonstrated a long-term risk for PVD. Screening of a cohort of extremely preterm infants with a history of moderate or severe BPD identified that 38% had PH [97]. This was resolved in the majority of patients at a median of 12.3 months (range 0.7–46.6 months). Interestingly, although pulmonary pressures in children with a history of PH had decreased and conventional markers had normalised over time, more sensitive measures such as the right ventricular longitudinal strain remained abnormal [97]. Takeoka et al. further observed that even after apparent resolution of ultrasound-defined PH, half of the patients had PH at 2 years of age, diagnosed by catheterisation [86].

Zivanovic et al. performed echocardiography as part of a longitudinal follow-up study on a group of preterm (< 29 weeks) children age 10–14 years, who were clinically well and demonstrated higher systolic pulmonary artery pressures in preterm children compared to term [87]. Two children had echocardiographic evidence of PH and required referral for clinical assessment [87].

A case series of prematurely born individuals in their 20 s reported evidence of early pulmonary vascular disease, including elevated pulmonary pressures and right ventricular dysfunction on right heart catheterisation [7]. These studies suggest that preterm adults are at increased risk for PVD throughout life, likely due to developmental disruption of lung and pulmonary vascular growth during infancy, highlighting the need for extended cardiovascular follow-up [98].

## Cardiorespiratory fitness

Cardiorespiratory fitness (CRF) is a strong and consistent predictor of morbidity and all-cause mortality in the adult general population [99]. Survivors of preterm birth have an increased risk of cardiometabolic morbidity (obesity, diabetes, hypertension), early COPD, and a fivefold increased risk of all-cause mortality from birth to 45 years of age [6, 100]. There is growing evidence highlighting reduced CRF in individuals born preterm [101–103]. Although assessment of CRF does not currently form part of the follow-up recommendations for preterm-born individuals with BPD as per ERS/ATS guidelines [11, 12], it should be noted that CRF is a modifiable risk factor that can be improved through increased physical activity and exercise training [104], and nearly half of the variance in CRF is attributable to habitual physical activity [105]. Lower self-reported physical activity has been reported in preterm-born individuals [103].

CRF is measured via cardiopulmonary exercise testing (CPET), which can evaluate respiratory, cardiovascular and metabolic physiological responses to increasing energy demands during exercise [106–108]. CPET interpretation is based on the principle that oxygen consumption (VO_2_) is the primary measure of exercise capacity [109]. CPET is most often reported using breath-by-breath gas analysis during exercise of increasing intensity on a treadmill or stationary bike [110]. Meta-analyses of CPET studies in preterm-born individuals have reported varied results and are limited by the relatively small amount and heterogeneity of the included studies [101–103]. In a meta-analysis of preterm-born children, preterms with or without BPD had a lower exercise capacity than term-born controls [101]. The authors reported that these differences were small, corresponding to a reduction in VO_2_ max of approximately 2–3 mL/kg/min [101]. Larger differences were seen in a more recent meta-analysis of adults born with very low birth weight, with significantly lower VO_2_max/VO_2_peak compared to controls [103]. Meta-analysis of studies solely reporting on those who had BPD found they were more likely to have a lower VO_2_max than term controls (mean difference, 6 mL/kg/min) [103].

A comprehensive meta-analysis of CPET outcomes including aerobic capacity, ventilation and gas exchange and cardiovascular and metabolic parameters [102] reported that at peak exercise, preterms demonstrated lower aerobic capacity, altered ventilatory and gas exchange capacity and reduced cardiovascular capacity compared to term infants. These results highlighted the complex cardiorespiratory limitations in this population who might benefit from targeting for rehabilitation.

Rehabilitation programmes are non-pharmacological treatments which can result in functional improvements in CRF [4]. In a randomised controlled trial of preterm children with BPD (aged 4–6 years), physical rehabilitation resulted in an improvement in the 6-min walk test. An important finding was the improved FEV_1_ score in the intervention group (mean FEV_1_% predicted: 102% pre-intervention versus 104% post-intervention) [111]. Although the quantified clinical impact may be limited, this finding conceptually emphasises that functional improvements can be achieved through exercise rehabilitation.

A randomised controlled trial of exercise to prevent hypertension in young adults compared a 16-week physical activity intervention consisting of structured exercise, motivational coaching and self-monitoring of physical activity with a control group receiving usual care. The subgroup analysis of preterm-born individuals who participated in the exercise arm showed an increase in the VO_2_ at the anaerobic threshold compared with preterm controls, an increase in the estimated cardiac index at the ventilatory threshold and an increase in minute ventilation at the ventilatory threshold [112].

## Suggested follow-up for extremely preterm infants and infants with BPD

Although some degree of respiratory morbidity might be expected in all preterm infants irrespective of a diagnosis of BPD [113], the current evidence base to inform specific recommendations for long-term cardiorespiratory follow-up is stronger for individuals born extremely preterm and those with moderate-severe BPD. The most studied methods include spirometry**,** echocardiography and cardiopulmonary exercise testing. These modalities, therefore, should form the core of the long-term assessment. Additional techniques—such as gas transfer, body plethysmography, lung clearance index, forced oscillation and imaging—show promise but require further longitudinal evaluation before they are routinely incorporated into clinical care.

In relation to the timings and duration of follow-up, it should be noted that puberty represents the last major physiological window for catch-up lung growth of the parenchyma and airways [55, 114]. Assessment immediately before and after puberty may help to further classify individuals to specific cardiorespiratory phenotypes. Finally, lung function typically peaks in early adulthood [115], making assessment at 24–26 years critical for determining the attained peak function and anticipated longer-term risk. From the content of this review, it follows that ideally follow-up of chronic neonatal respiratory disease should be a lifelong undertaking; we do recognise, however, realistic constraints relating to lack of trained personnel and associated costs for the families and the health system. We thus propose four key assessment time points beyond the early childhood follow-up (2 years corrected age) for individuals born extremely preterm (≤ 28 weeks) or with moderate-severe BPD: childhood (6–8 years), pre-pubertal (11–14 years), post-pubertal (16–18 years) and young adulthood (24–26 years). Since spirometry and CPET are generally not feasible before 6 years, a comprehensive cardiorespiratory assessment including exercise assessment at 6–8 years is important as a first baseline assessment of cardiorespiratory health. Individuals with severely abnormal parameters or deteriorating cardiorespiratory function should remain in structured follow-up for as long as these abnormalities persist, bearing in mind the possible impact of the natural lung function decline on respiratory health in this population.

Although this review was mainly focused on the cardiorespiratory aspect of the follow-up, there is growing evidence that chronic lung disease of prematurity is associated with multi-systemic comorbidities, and hence even more comprehensive follow-up programmes might be required, including assessment of extrapulmonary morbidities and targeted lifestyle interventions [116].

## Conclusions

In conclusion, chronic respiratory morbidity following premature birth is associated with significant, often lifelong complications affecting all aspects of cardiorespiratory health. There is currently wide variation in the follow-up practices and limited evidence to inform follow-up recommendations. We suggest that follow-up of these infants should extend to early adulthood and focus on capturing and describing not only lung function test abnormalities, but also including cardiorespiratory exercise testing and assessment for pulmonary vascular and parenchymal disease.

## Data Availability

No datasets were generated or analysed during the current study.
